# Modern Treatment of Supracondylar Humeral Fractures in Children

**DOI:** 10.3390/children12050556

**Published:** 2025-04-25

**Authors:** Adrian Surd, Rodica Muresan, Carmen Iulia Ciongradi, Lucia Maria Sur, Lucia Raluca Ardelean, Lia Oxana Usatiuc, Kriszta Snakovszki, Camelia Munteanu, Ioan Sârbu

**Affiliations:** 1 Pediatric Surgery and Orthopedics, “Iuliu Hațieganu” University of Medicine and Pharmacy Cluj-Napoca, 400347 Cluj-Napoca, Romania; adisurd@elearn.umfcluj.ro; 2 Pediatric Surgery and Orthopedics, Emergency Children Hospital Cluj-Napoca, 400177 Cluj-Napoca, Romania; muresanrodicaana@elearn.umfcluj.ro (R.M.); ardelean_lucia_raluca@elearn.umfcluj.ro (L.R.A.); kriszta.sztrelenczuk@elearn.umfcluj.ro (K.S.); 3 Pediatric Surgery and Orthopedics, “Grigore T. Popa” University of Medicine and Pharmacy, 700114 Iasi, Romania; carmen.ciongradi@umfiasi.ro; 4 Pediatrics 1, “Iuliu Hațieganu” University of Medicine and Pharmacy Cluj-Napoca, 400347 Cluj-Napoca, Romania; sur.maria@umfcluj.ro; 5 Pathophysiology, Department of Functional Sciences, “Iuliu Hațieganu” University of Medicine and Pharmacy Cluj-Napoca, 400347 Cluj-Napoca, Romania; 6 Biology Section, Faculty of Agriculture, University of Agricultural Sciences and Veterinary Medicine Cluj-Napoca, 400372 Cluj-Napoca, Romania; camelia.munteanu@usamvcluj.ro; 7 2nd Department of Surgery—Pediatric, Surgery and Orthopedics, “Grigore T. Popa” University of Medicine and Pharmacy, 700114 Iasi, Romania; sarbu.ioan@umfiasi.ro

**Keywords:** supracondylar, children, treatment

## Abstract

Supracondylar humeral fractures are the most common type of elbow fractures in children. The treatment methods vary depending on the type of fracture (Gartland classification), which can be conservative or surgical. There is no clear consensus or guidelines to dictate the treatment of complicated supracondylar humeral fractures (Gartland types II and III). Gartland type II and III fractures are most frequently treated with closed reduction and percutaneous Kirchner-wire pinning or open reduction with K-wire pinning, depending on the degree of displacement and the orthopedic surgeon’s preference. Most studies recommend avoiding open reduction because of prolonged hospitalization and higher rates of complications. Orthopedic surgeons have different opinions regarding the Kirschner pin placement technique. Studies suggest that only lateral pinning is safe and effective, but medial and lateral pinning is proven to give more stability; there is always a risk of iatrogenic ulnar nerve damage during surgery. Modern treatment of supracondylar humeral fracture in children should focus on minimally invasive techniques and avoid open reduction, when possible, to ensure the best outcome for the patients. This scoping review’s purpose is to gather the available information on the topic in one place and to underline the lack of clear protocols.

## 1. Introduction

Supracondylar humeral fractures are the most common type of elbow fractures in children [1]. The median age of presentation is six years [2,3,4]. Incidence steadily declines until age 15, at which point patients typically have an adult pattern [4]. Although there is conflicting evidence, some reports suggest that females are more likely to sustain this injury [5,6], and a recent analysis of a cohort of more than 63,000 children over five years failed to find a statistically significant difference [7]. A fall onto an outstretched hand, with the axial transmission of body weight through the maximally extended elbow, is typically the mechanism of injury. Due to the ligamentous laxity prevalent in this age range, elbow hyperextension may be impacted by this, resulting in an extension-type fracture, which accounts for 97–99% of injuries [8,9]. More specifically, Herdea et al.’s 2021 study [10] found that the non-dominant hand is used more frequently during preschool (ages 4–6). This trend persisted and even increased during the 7–14 age range. This study concluded that children often fall on their non-dominant hand to protect their dominant hand [10]. Moreover, inadequate levels of 25(OH)D increase the risk of bone fragility and fractures [11]. The treatment methods vary regarding the type of fracture (Gartland classification), from casting in non-displaced fractures to the treatment of complicated supracondylar humeral ones (Gartland types II and III), which usually need closed reduction and percutaneous K (Kirschner) wire placement. The treatment method is generally up to the orthopedic surgeon and the hospital protocol. The elbow has a lot of sensitive anatomical structures, like the ulnar nerve and radial artery, which can become injured by the trauma itself or during surgery. This scoping review’s purpose is to analyze and synthesize the current literature regarding the optimal treatment strategy for Gartland type II and III supracondylar humeral fractures in children, concentrating on the efficacy and safety of minimally invasive techniques, especially different Kirschner-wire pinning methods. Because of the lack of a consensus, this work intends to clarify treatment protocols that minimize complications and improve outcomes.

## 2. Methods

### 2.1. Search Strategy

**Figure 1:**
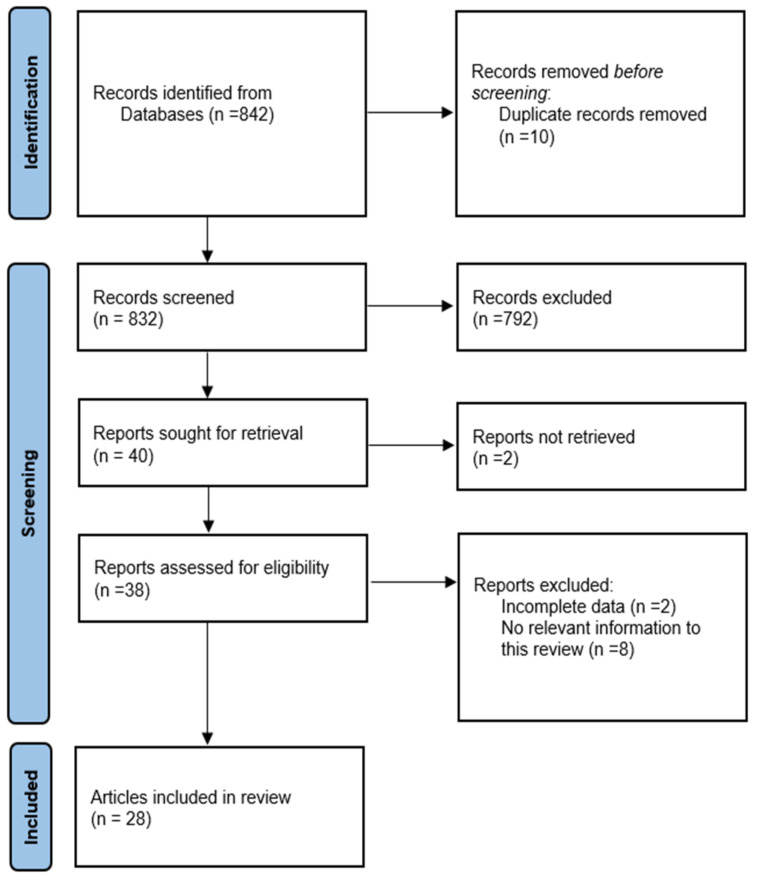
PRISMA flow diagram.

The articles cited in this narrative review were collected from two databases, PubMed MEDLINE and Google Scholar, using the following keywords: supracondylar, children, and treatment. We selected studies that were published in the last 10 years. We identified 842 records through database searching. After removing the duplications (10 articles) and reading the abstracts, we selected 40 reports. After excluding 12 articles with reasons, we selected 28 articles to include in this paper. A comprehensive flowchart for the selection of studies is depicted in Figure 1.

### 2.2. Risk of Bias Evaluation

Multiple reviewers independently assessed each article, and any discrepancies were resolved through discussion to reach consensus. By systematic research and multiple reviewers, the likelihood of selection and reporting bias was reduced, enhancing the overall reliability of the findings in this review. Consensus was reached among all researchers regarding the selected studies.

## 3. Results and Discussion

### 3.1. Anatomical Considerations in Pediatric Elbow Fractures

The elbow joint is composed of the distal end of the humerus (capitulum and trochlea), the proximal end of the radius (radial head), and cubitus (olecranon fossa) bones. The capitulum and the olecranon fossa form the active part of the joint. Depending on age, the capitulum and the olecranon head are not fully developed in children. Therefore, it is essential to know the radiological aspect of the bones for each age category [1].

Also, the median, radial, and ulnar nerves, as well as the brachial, radial, and ulnar arteries, are vital near the elbow. Damage to these structures from supracondylar fractures may result in issues with blood flow or nerve function. Moreover, the brachial artery and the periosteal vessels supply blood to the humerus. A fracture may interrupt this supply, which could impact healing. Blood flow must be maintained throughout treatment [12]. Consequently, the anatomical relationships of the arteries and nerves surrounding the elbow, as well as the nutrient supply to the epiphysis, should be carefully considered when treating children’s supracondylar humeral fractures. These elements impact both the healing process and the therapeutic approach used.

Supracondylar humeral fractures are the most common type of elbow fracture in children [1]. Depending on the degree of the fracture (Gartland classification), treatment can vary from immobilization in a cast system to surgery, which can be minimally invasive (closed reduction and percutaneous pinning) or open [13].

The supracondylar region in the pediatric age is fragile due to variations in microarchitecture [14]. Also, the anteroposterior and lateral diameters are shorter when compared to the adult distal humerus, the cortex is thinner, the distal epiphysis is more cartilaginous, and the olecranon and coronoid fossa are wider; ligamentous laxity is also present, all of which contributes to the mechanism of the supracondylar humeral fracture [14].

### 3.2. Evaluation of Supracondylar Humeral Fractures

#### 3.2.1. Fracture Classification

Supracondylar humeral fractures are classified according to the mechanism, displacement severity, and lesion level. There are two types of fractures: extension and flexion.

Extension-type fractures are the most common, accounting for more than 95% of the cases. This lesion usually occurs when the child falls with the elbow in hyperextension, and the olecranon is forced into the olecranon fossa, resulting in a fragment displaced posteriorly. Flexion-type lesions are rare (around 5%), and in this kind of mechanism, the olecranon acts as a fulcrum, pushing the distal fragment anteriorly [15,16,17].

**Figure 2:**
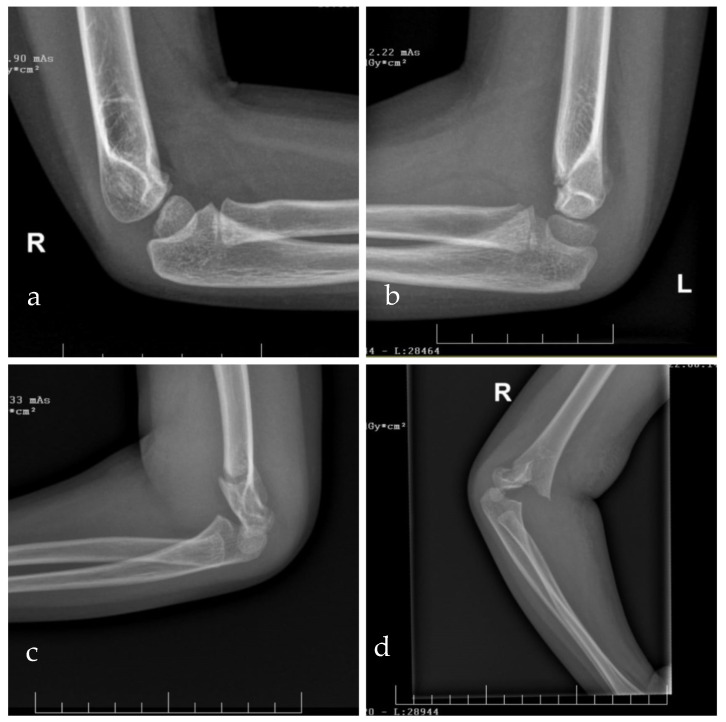
Radiographs of supracondylar humeral fracture according to Gartland classification modified by Wilkins, from our patient cohort. Gartland type I (**a**), Gartland type II a (**b**), Gartland type II B (**c**), and Gartland type III (**d**).

In 1959, Gartland et al. categorized supracondylar humeral fractures into three types according to their displacement. In 1997, Wilkins et al. completed the classification based on his observational studies [18]. Table 1 provides a summary of the supracondylar humeral fracture classification, adapted from Wilkins et al. [19]. Figure 2 illustrates representative radiographic examples of supracondylar humeral fractures, categorized according to the Gartland classification.

Based on the above classification, treatment ranges from conservative with cast immobilization to closed or even open reduction and Kirchner wire placement. Type I and type IIA fractures are usually treated with immobilization in a cast system, and types IIB and III require surgical treatment, closed or open reduction with K-wire pinning, depending on the orthopedic surgeon’s choice and hospital protocol [13,19].

In 2006, Leitch et al. described a special kind of fracture, the complete rupture of the periosteum hinges, lacking support at the broken end and extreme instability in the extension and flexion of the joint [20]. This type of fracture is found in 3% of the cases, and it was classified as a Gartland type IV fracture (multidirectional unstable supracondylar humeral fracture). Most flexion-type fractures can be considered Gartland type IV [20].

Supracondylar fractures can also be evaluated by the level of the fracture line in low or high fractures reported to the distal humeral isthmus [21]. A low fracture type was associated with higher chances of poor clinical outcome because of the juxta-articular nature of the fracture and the small bone fragment, which can affect the joint and the ligaments and is recognized as a cause of elbow stiffness [21].

Nevertheless, this type of fracture can be an open or closed fracture, depending on the mechanism of the injury [1]. However, supracondylar humeral fractures can be categorized based on the level of the fracture line about the distal humeral isthmus as well as the mechanism of injury (whether open or closed) [22]. Since low fractures are closer to the joint, there is a greater chance of poor clinical results because of the small bone fragments and how they affect the ligaments and joints, which can cause elbow stiffness. The degree and kind of fracture significantly impact the course of treatment and long-term prognosis, regardless of the cause (open or closed) [22].

#### 3.2.2. Clinical Evaluation

Usually, the child presents in the emergency department with pain, impotence, local deformity, and sometimes with vascular or neurological impairment [1].

Vascular injuries can be evaluated at initial presentation by palpating the radial pulse, measuring the capillary refill time, and assessing the skin distal to the fracture [23]. If the skin in the cubital fossa is depressed and shows ecchymosis, it can be a sign of a displaced supracondylar humeral fracture, and the probability of the brachial artery being trapped in the fracture site is higher. This may lead to transection, kinking of the artery with reduced flow, thrombosis of the vessel, or traumatic aneurysm of the brachial artery [23].

A thorough neurological examination must be conducted, sometimes in an informal way, especially in a young patient [13]. The motor function can be evaluated by a “rock, paper, scissors, OK” game; the median nerve can be assessed by asking the child to make a fist (rock), the radial nerve by extending the fingers (paper), and the ulnar nerve by abducting the fingers (scissors). The anterior interosseous nerve can be evaluated by asking the patient to flex the thumb’s interphalangeal joint and the index’s distal interphalangeal joint (OK sign). It is essential to also consider the sensitive function of the nerves by assessing sensation and documenting if we find deficits [13]. The most commonly injured nerve is the anterior interosseous (median nerve), followed by the radial nerve [24].

#### 3.2.3. Radiological Evaluation

Antero-posterior and lateral radiographs are usually performed to highlight the fracture site. In Gartland type I fractures (non-displaced and stable fractures), the only radiological sign that may be present is the effusion signified by an anterior or posterior “fat pad sign” with a 75% positive prediction of an occult fracture [13].

The following radiographic parameters can be assessed for a complete evaluation of the fracture [14]:

- Baumann angle (BA): the angle between the axis of the humeral diaphysis and the line parallel to the fossa of the lateral condyle, standard value 64–81—it is representative of the coronal plane dislocation.
- Antero-humeral line (AHL): the parallel line drawn to the humerus that intersects the middle third of the capitulum nucleus—it is representative of the sagittal plane dislocation.
- The carrying angle.
- Humeroulnar angle.
- Metaphyseal–diaphyseal angle [14].

These parameters can be calculated preoperatively and postoperatively in follow-up to predict the patient’s clinical outcome. The Baumann angle and AHL were extensively studied to assess the functional outcome after surgical treatment [14].

The normal values of each angle and the incidence on which it is measured (AP or lateral) are explained here:

**Anterior Humeral Line (AHL):**

Normal: In the side view, the line should pass through the center of the capitellum, the spherical portion of the elbow.

**Capitellum-Coronoid Line:**

Normal: In the side view, the line should go through the center of the humerus, or upper arm bone.

**Carrying Angle:**

Normal: When viewed from the front (AP view), the elbow angle should be between 11 and 15 degrees.

**Baumann’s Angle:**

Normal: From a frontal perspective, about 64–81 degrees, with a mean value of 75 [25].

### 3.3. Management Strategies

#### 3.3.1. Non-Surgical Treatment

Gartland type I and IIA supracondylar humeral fracture treatment consists of immobilization (long cast at 60–90° flexion, neck sling) for 3–4 weeks [5]. After the treatment, the patients are usually clinically and radiologically evaluated. If no complications exist and the X-ray shows sufficient callus formation, the child should start active elbow movements [5]. Type I fractures are defined as stable fractures, with no deviation of the AHL or a minimal deviation of 2 mm [5]. Usually there is no need for surgical treatment. Still, a common pitfall is not recognizing medial impaction, which could result in varus deformity and not correct with growth. If medial compression is present, a closed reduction should be performed using external maneuvers under general anesthesia [24].

Wang et al. recommend appropriate exposure to sunlight during healing time (at least 2 h every day). If not possible, vitamin D supplements should be prescribed to the patients to promote healing of the bones. Vitamin D plays a vital role in growth plate-mediated endochondral osteogenesis and osteoblast-mediated bone synthesis, and sunlight is the primary endogenous method to obtain vitamin D [26]. Furthermore, the research underscores the importance of regular medical consultations to ensure optimal 25(OH)D levels in children, potentially necessitating updates to current sufficiency guidelines. Addressing 25(OH)D insufficiency promotes children’s musculoskeletal health and well-being [27].

According to Thomas et al., using Blount’s method, almost all kinds of supracondylar humeral fractures can be managed non-operatively. Blount’s method consists of fracture manipulation under a mild sedation, which can be performed in the emergency room. The patient’s arm is positioned supine, and under fluoroscopic guidance, extension, flexion, pronation, and supination movements are made to reestablish bone continuity. After the procedure is performed, a cuff and collar immobilization can be applied on the patient’s limb at a 120-degree angle. In the above-mentioned study, 87.5% of the subjects healed without significant complications [28]. Muccioli et al. conducted a study to determine if Blount’s method can be considered a first-line treatment in supracondylar fractures. Almost all patients enrolled in his study had satisfactory outcomes according to Flynn’s criteria [29].

#### 3.3.2. Surgical Treatment

##### Closed Reduction with Percutaneous Kirschner-Wire Pinning

Zou et al. suggest that the optimal time to perform surgery is as soon as possible to avoid complications, such as significant edema, which can result in compartment syndrome. In some cases, surgery cannot be performed at presentation (anesthesia protocol, technical issues, and significant swelling on presentation). The delay of surgery was demonstrated to increase the chances of conversion to open surgery. It is generally accepted to be a 1–3-day delay. Children should benefit from a loose cast if the surgery cannot be performed immediately [30].

Gartland type IIB and III fractures are usually treated with closed reduction under fluoroscopy and percutaneous pinning of Kirschner wires and cast immobilization for 4 weeks [31]. Surgeons’ opinions are divided when it comes to the placement of K-wires [30]. Dislocated type IIA fractures are an exception—they can be treated with or without closed reduction and cast immobilization. Still, if the stability of the fracture is not guaranteed, the distal fragment should be stabilized by percutaneous K-wire pinning [31].

Medial displacement of the distal fracture was identified to be more common than lateral displacement, occurring in approximately 75% of cases. This displacement type puts the radial nerve at risk, while posterolateral displacement affects the median nerve and brachial artery [31].

In some cases, when surgery needs to be delayed, a closed reduction in general anesthesia and casting may be an option to promptly re-establish the length of the humerus, release entrapped soft tissues, alleviate muscle spasms, and ultimately relieve the pain [30]. Patients who underwent closed reduction and temporary casting before definitive repair had a better surgical outcome, lower pain rates even post-surgery, and required fewer analgesics [30]. Zou et al. conducted a study in which patients were assigned to two groups: the first group had surgery close to presentation time, and the second group benefited from closed reduction and temporary cast placement first. The two groups had no significant differences regarding Flynn grade or change in Baumann angle [30].

**Figure 3:**
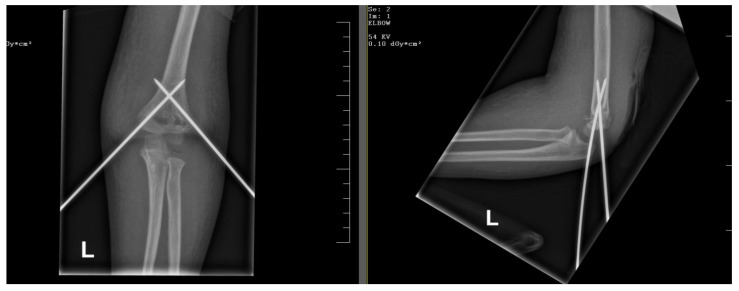
Cross-pinning of the K-wires in a Gartland type III fracture.

More studies suggest that cross-pinning of the surgical material gives better biomechanical stability of the fracture, but there is a higher chance of iatrogenic ulnar nerve damage. The frequency of iatrogenic ulnar nerve damage ranges from 1.4% to 15.6% [31,32,33]. Many physicians and authors preferred the lateral pin configuration because of the possibility of iatrogenic nerve injury during the cross-pinning procedure [31] (Figure 3). Minifee et al. studied the outcomes of closed reduction and medial percutaneous pinning, and his results were satisfactory, with no complications at three months follow-up [34]. An adequate alternative for the medial cross-pinning is the Dorgan procedure [35]. The components of the Dorgan procedure are closed reduction and lateral pinning of the K-wires in a cross configuration. The first pin is inserted through the lateral condyle across the fracture into the medial cortex. The second wire is introduced through the lateral cortex, close to the fracture line, and driven into the medial condyle in an antegrade direction, stabilizing the fracture site. If closed reduction fails or the clinical presentation is suggestive (open fracture), open reduction is an alternative [35]. This method decreases the chances of ulnar nerve injury and assures better stability of the fracture, promoting healing. Because of the high entry point of the second wire, there is a risk of radial nerve injury, but with rigorous technique this complication can be avoided [35].

Lateral pin configuration is shown to have poorer stability, but there is a more negligible risk of iatrogenic ulnar nerve injury during pin placement. Santosh et al. suggest that lateral pin entry cannot fully stabilize the proximal end of the fracture, making the construct biomechanically unstable [32]. Some studies suggest that divergent lateral placement of the Kirschner pins could decrease stability at the fracture site [36]. Segal et al. found that fracture obliquity could be a factor for loss of reduction, especially in lateral pinning. He also demonstrated that Gartland classification correlated with sagittal plane obliquity (*p* < 0.001) but was statistically insignificant when tested as an indicator for loss of reduction [36]. However, many surgeons prefer lateral entry pinning because it can achieve sufficient stability while eliminating the risk of ulnar nerve injury. Still, the fracture line should configure the pins to achieve the best results [37]. Xianglu et al. conducted a two-stage retrospective analysis to determine the effect of the proximal pin’s entry and exit points in lateral pinning. They concluded that the entry and exit points were not statistically significant, which should be as high as possible to achieve better stability [37,38].

Pavone et al. conducted a study to determine if the prone position of the patient on the operating table is superior to the supine position [33]. Prone positioning can help in posterior displacement fractures; the gravity force correctly positions the distal humerus in the coronal plane and, more importantly, avoids elbow hyperflexion, which is generally needed in patients treated in the supine position. During elbow hyperflexion, the ulnar nerve slips anteriorly out of the cubital tunnel, passing over the medial epicondyle; this can increase the risk of iatrogenic nerve damage during medial pinning. On the other hand, the supine position allows standard anesthesia management and the possibility of using anterior approaches if necessary. The authors concluded that the supine position is not superior to the prone position in surgical technique [33].

More authors and surgeons tried to hypothesize a complementary method to aid closed reduction in complicated humeral fractures (Gartland type IV or flexion type fractures) [39]. This kind of fracture needs anatomical reduction, ideally in a single intervention. They tried lever-assisted closed reduction and used a blunt-tip K-wire as a lever, which was introduced through soft tissue to the anterior and posterior cortex of the fracture fragment. Some authors labeled this technique the ‘Joystick’ method. A successful reduction is influenced by factors such as the robust and elastic brachialis muscle and fascia enveloping the distal humerus and joint, the anterior periosteum of the bone, and the impaction of periosteal soft tissues [39]. After introducing the blunt Kirschner wire, the assistant maintained the lever in position. The surgeon introduced three Kirschner wires laterally; the radial pulse was checked constantly during this procedure. This study’s results were satisfactory; all patients had good outcomes according to Flynn’s criteria [20,40]. Lin et al. hypothesized a technique for lever-assisted reduction in which the de-sharpened K-wire is drilled into the medullary cavity of the distal fracture fragment [41]. The newly created lever acts with an elastic force, which enhances the reduction in the number of severely displaced humeral fractures, especially if the proximal fragment is rotated [41]. This method can obtain a better surgical approach, the stability of the fracture will not be lost during C-arm fluoroscopy, and it also reduces manual reduction times and corrects the Baumann angle more effectively. Additionally, this method causes minimal disruption to the blood supply, reducing disturbance to the joint structures, which results in faster healing, optimal recovery of the elbow function, and minimal scarring [39,42].

The fixation with elastic stable intramedullary nail after closed reduction was described. This kind of surgical repair’s advantage includes cast-free treatment after surgery and protection of the ulnar nerve by introducing the nail at the proximal humerus. In complicated supracondylar fractures, it is rarely suggested to avoid cubitus varus due to the necessity of anatomical reduction. It is not feasible for a Gartland type III/IV [32].

In some cases, external fixation can be considered a treatment option [42]. An external fixator provides considerably more stability than percutaneous pinning, and casting is not used in this case. However, in our experience, this immobilization can be uncomfortable for pediatric patients [43].

Pulseless supracondylar humeral fractures can occur in 3–19% of cases in severely displaced fractures [23]. The distal pulse and capillary refill time should be evaluated bilaterally upon presentation. A clinical occurrence is the pink hand but with a non-palpable radial pulse; in this scenario, a Doppler ultrasound can be used to detect the presence of distal perfusion. These borderline cases can be managed conservatively by loose splinting in less than 90 degrees flexion and closed monitorization to promptly identify a late-developing vascular compromise [23]. Surgical management consists of closed reduction and internal fixation, often leading to pulse and perfusion restoration. In some cases, closed reduction does not restore blood flow, and open exploration is required. Surgeons should be aware of the possibility of interposed structures at the fracture site when a pulseless supracondylar fracture poses a difficult reduction or a “spongy” feel with a residual anterior gap at the fracture site visualized on fluoroscopy. Such interposition should be considered when vascular signal is lost after closed reduction and fixation and should prompt pin removal and open exploration via an anterior approach [23].

Surgeons who advocate for closed methods of reduction with K-wire pinning state that few complications have occurred, such as loss of movement or infection of the pin site. Also, patients with closed reduction had a shorter hospitalization time than those who underwent open reduction [44].

Pesenti et al. conducted a study in which he made a comparison between five types of closed reduction and percutaneous pinning. The five methods were stable intra-medullary nailing, two pins in an X configuration, two lateral pins and a medial pin, two lateral pins, and three lateral pins. He observed that immediate instability was not statistically significant across the five groups. Also, he concluded that medial pinning was less likely associated with secondary displacement (*p* = 0.04) but did not affect the risk of nerve damage [45].

##### Open Reduction with Percutaneous Kirschner-Wire Pinning

In some cases, minimally invasive surgery is not feasible [19]. Occasionally, severely displaced supracondylar humeral fractures are challenging to reduce due to the proximal fragment buttonholing through the brachialis muscle, interposition of the capsule or the periosteum, or interposition of neurovascular structures (median nerve, ulnar nerve, brachial artery). Anatomical reduction should be the goal to avoid early and late complications [19,32].

Some authors suggest that repeated closed reduction is not recommended, as it may lead to iatrogenic soft tissue injury [20]. If closed reductions fail, an open reduction should be considered [20,31]. Also, open fractures should be approached by open reduction and percutaneous K-wire pinning [31].

Nevertheless, open reduction led to concerns regarding elbow stiffness, myositis ossificans, more extended time of union, reduced range of movement, unsightly scarring, and iatrogenic neurovascular injury [39,40,43], but these complications can also occur in multiple failed closed reduction attempts [44]. Hussein et al. conducted a study comparing the functional outcomes of children treated with open reduction versus closed reduction. The results by Flynn criteria were 84.8% in the open reduction group and 90.9% in the closed reduction group [44].

Different studies have drawn conflicting conclusions regarding the optimal moment for surgery [46]. Some authors concluded that delay of the surgical intervention for more than 24 h in flexion-type supracondylar humeral fractures was shown to increase the chances of converting to open surgery. Other researchers found that a delay ranging from 36 h to 7 days did not increase the necessity of open surgery [46].

In rare cases, open fractures can occur in children. Open fractures are categorized using the Gustilo–Anderson classification. Depending on the severity of the injuries, a thorough washout of the wound is necessary, followed by antibiotic treatment and surgery. Gustilo type I, II, or IIIA fractures do not always need surgical intervention [47].

Open reduction followed by percutaneous pinning can be considered a valid first-line treatment for type III and IV supracondylar humeral fractures in the presence of several complex injuries (severely displaced open fractures, multidirectional unstable fracture site, vascular or neurological impairment). Some open fractures, like Gustilo type I, can be treated with closed reduction and percutaneous pinning [19].

### 3.4. Complications and Post-Treatment Evaluation

Complications can be classified as immediate or delayed complications. Immediate complications include nerve damage, arterial blood flow impairment, and compartment syndrome [23,48]. Varus or valgus deformity, loss of range of motion, stiffness [32,49], Volkmann ischemic contracture, and, in some rare cases, trochlea necrosis [23,26] can be delayed complications.

After treatment, the child needs to be evaluated thoroughly. This is usually carried out by conducting a thorough physical exam and X-rays. On the physical exam, the orthopedic surgeon should focus on clinical appearance and range of motion and always compare it to the healthy limb to determine neurological impairment [30]. In most cases, neurological dysfunction is temporary and will resolve spontaneously [30]. Parameters such as AHL, Baumann angle, and tilting angle can be obtained by performing X-rays [25]. These parameters are included in Flynn’s criteria, a valuable tool for assessing supracondylar humeral fracture treatment’s functional and cosmetic outcomes [42]. Flynn’s criteria evaluate the treatment’s total effectiveness by combining functional and cosmetic results. The two main components of the criteria are functional deformity and cosmetic deformity.

Cosmetic Deformity: This describes how the arm looks after therapy, such as any discernible elbow misalignment or angular deformity.

Functional Deformity: This assesses how the fracture impacts the child’s arm use, specifically elbow range of motion.

An orthopedic surgeon’s goal should be anatomical reduction and good clinical outcomes. Knowing the pattern of bone growth is essential. Only 20% of the final upper arm and forearm length comes from the elbow growth plates, mainly in the first years of life. Healing is usually complete in children under 5, while bone malalignment can happen in older children [49].

The following table (Table 2) summarizes the information extracted from significant studies.

### 3.5. Study Limitations

This article has its own limitations. The article number is modest but very rich in information. Since it is a literature review, we did not statistically analyze the information, but we rather gathered it to summarize the known knowledge at this moment on the selected topic.

## 4. Conclusions

Supracondylar humerus fractures in children account for 70% of elbow fractures. They are considered the second most common type of fracture in children around 7 years of age, accounting for 16.6% of fractures in this age range [44].

To summarize the information extracted from the above-cited articles, treatment for Gartland type I fracture consists of casting; Gartland type IIA also benefits from casting and Gartland type IIB, depending on the clinical presentation, can benefit from casting or closed reduction with percutaneous pinning. Gartland types III and IV require surgical treatment, which consists of closed reduction and Kirschner wire pinning or, in selected cases, open reduction.

However, surgeons worldwide often disagree on how to apply reduction procedures properly. Notably, disagreement exists on the best ways to pin and to select the proper technique, regarding the severity of the fracture. Additional study and technique standardization may enhance the clinical and functional outcomes for children with supracondylar fractures, as this lack of homogeneity may affect the outcomes. Treatment should focus on minimally invasive techniques when possible. Nevertheless, it is essential to aim for the best clinical and functional outcomes, which can be achieved with transparent and standardized protocols.

Considering the foregoing, we recommend developing and implementing fracture prevention strategies following a fall. These strategies should minimize the instinctive tendency to favor the non-dominant limb and, where practicable, promote the equitable distribution of stress across both limbs during impact. Such measures can be incorporated into both individual and group sports-related activities [10].

## Figures and Tables

**Table 1:** Modified Gartland classification of supracondylar humerus fractures.

	Gartland Classification Modified by Wilkins
Type I	Non-displaced
Type II	IIA—Minimally displaced; posterior cortex is intact IIB—Rotational or non-rotational displacement, but in contact
Type III	Totally displaced with posterolateral or anteromedial fragment; no contact
Type IV	Most are flexion-type fractures; multidirectionally unstable supracondylar humeral fractures

**Table 2:** Supracondylar humerus fractures in pediatric patients: treatment modalities and outcomes.

Study	Number of Subjects	Closed Lateral Pin Configuration	Closed Medial Pin Configuration	Open Reduction	Conservative Treatment	Complications —Number of Subjects	Functional Outcome by Flynn Criteria
Dučić et al. [31]	93	0	37	34	22	Neurological complications—4 Cubitus varus—3 Elbow stiffness—2	Excellent—69 Good—20 Fair + poor—9
Banshelkikar et al. [32]	100	47	53	3	0	Pin tract infection	Excellent—93 Good—5 Fair—2
Pavone et al. [33]	59	59	0	0	0	Ulnar nerve paresthesia—2 Asymmetry—1 Varus deviation—1 Mild hyperextension—1 Local infection—1 Spontaneous removal of K-wires—1	Excellent—57 Good—2
Hannonen et al. [38]	195	19	141	5	30	Loss of reduction—7	**-**
Hussein Al-Algawy et al. [44]	66	33	0	33	0	Second trial reduction—6 Pin tract infection—3 Varus deviation—1 Ulnar nerve neurapraxia—1 Cubitus varus (20 degrees)—1	Excellent—21 Good—7 Fair—3 Poor—1

## Data Availability

No new data were created or analyzed in this study. Data sharing is not applicable to this article.

## Abbreviations

The following abbreviations are used in this manuscript:

MDPI	Multidisciplinary Digital Publishing Institute
BA	Baumann angle
AHL	Antero-humeral line
K-wire	Kirschner wire
